# New spontaneous breast seroma 5 years after augmentation: a case report

**DOI:** 10.4076/1757-1626-2-7126

**Published:** 2009-09-02

**Authors:** Danai Chourmouzi, Trantafillos Vryzas, Antonios Drevelegas

**Affiliations:** 1 Diagnostic Radiology Department, Interbalcan Medical CenterThessalonikiGreece; 2 Plastic Surgery Department, Interbalcan Medical CenterThessalonikiGreece; 3 Diagnostic Radiology Department, Interbalcan Medical CenterThessalonikiGreece

## Abstract

The case of a 36 year old woman who experienced a late, spontaneous breast seroma 5 years after augmentation in the absence of any known precipitating factors is reported. Although seroma is not an uncommon complication in the immediate postoperative period, it is extremely rare as a late complication of breast implantation. Magnetic resonance imaging is a reliable method to confirm the diagnosis of late seroma formation. Surgery is the preferred treatment.

## Introduction

Breast augmentation is the most common cosmetic surgery in the United States, according to the American Society of Plastic Surgeons. Breast implants are also used to reconstruct the breasts after mastectomy. There has been an increased incidence of breast augmentation in the last decade. Despite the widespread use of silicone gel breast implants, the prevalence of implant complications is unknown. General complications include capsular contracture, rupture, leakage, infection, and migration of the implant. Numerous cases describing both implant rupture and gel migration beyond the capsule have been reported in the literature [1,2].

Breast seromas are tumor-like collections of fluid in breast tissue that occur following excisional biopsy, lumpectomy, mastectomy, and plastic surgery procedures such as breast augmentation, prosthesis explantation, breast reduction, and breast reconstruction. Although seroma is not an uncommon complication in the immediate postoperative period, it is extremely rare as a late complication of breast implantation [3,4]. We present a new case of spontaneous breast seroma that presented 5 years after augmentation mammaplasty in the absence of any known precipitating factors. The role of MRI in the diagnosis is emphasized.

## Case presentation

A 36-year-old-Greek woman with bilateral breast augmentation performed 5 years previously presented with enlargement of her left breast. The woman underwent bilateral breast augmentation with retropectoral muscle implantation of textured silicone gel prostheses. The implants were inserted through inframammary incisions and the patient’s perioperative course was uneventful. Her past medical history was clear.

The enlargement was evident upon clinical inspection without other signs or symptoms of inflammation. Physical examination showed a swollen and tense left breast, which was very tender upon palpation. She was afebrile with no redness and no axillary lymphadenopathy. Her laboratory findings were normal.

**Figure 1.**
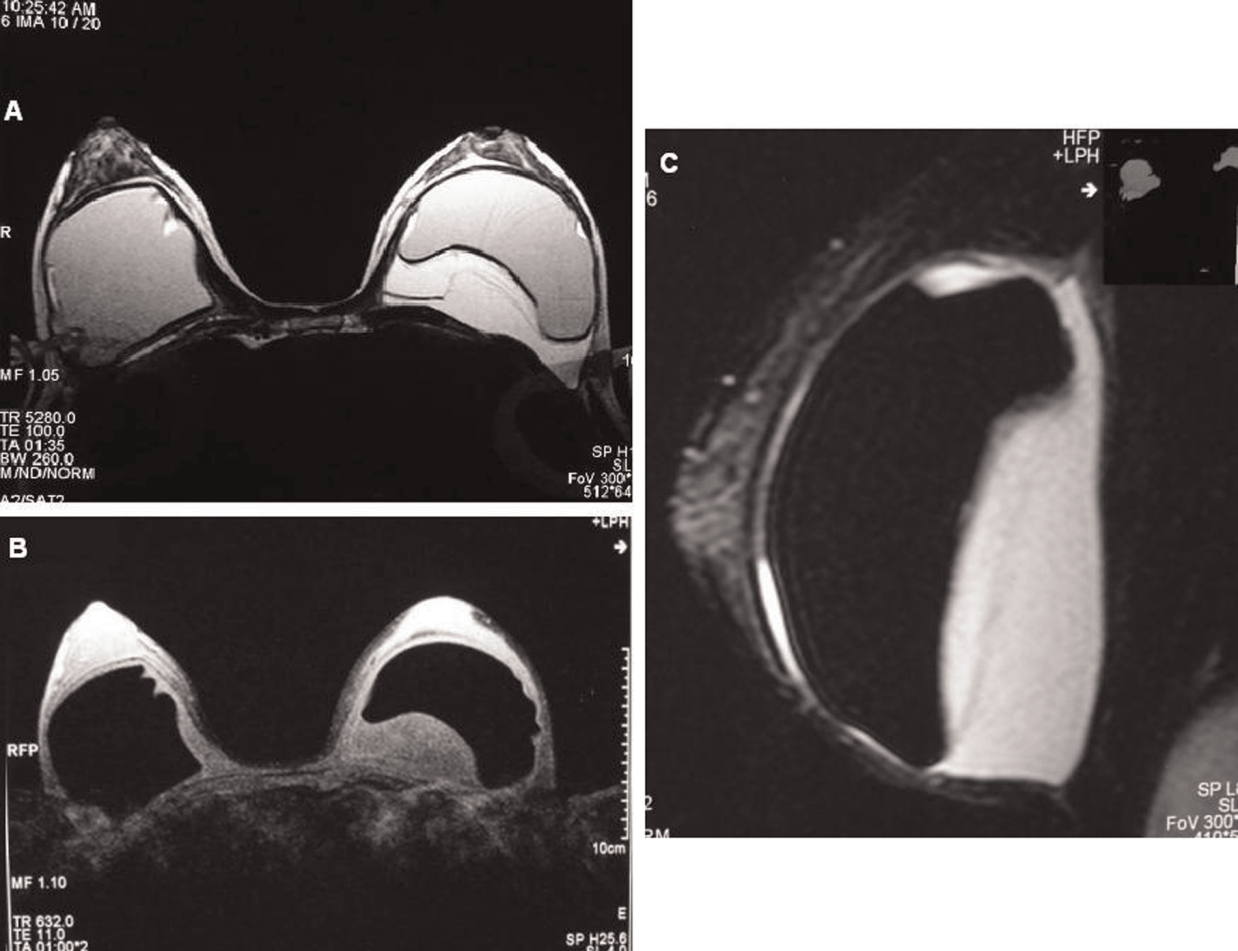
MRI examination of a retromammary seroma. **(A)** Axial T2-weighted image showing retroprosthetic collection with high signal intensity in the left breast. The silicone implant is intact. **(B)** Axial T1, with fat and silicone suppression, showing seroma with low signal intensity. Note the normal appearance of the right breast implant. **(C)** On sagittal STIR image with silicon suppression, the fluid collection shows high signal intensity.

An MRI examination was ordered. The goals of the MRI were to determine whether the implants were ruptured and whether any extracapsular silicone was present. We scanned the patient on a 1.5 T scanner with breast coil. Axial T2-weighted fast-spin echo sequence, axial T1-weighted sequence with fat and silicone suppression, and sagittal STIR with silicon suppression were obtained (Figure 1). A retroprosthetic fluid collection with low signal intensity on T1-weighted images and high signal on T2-weighted images were seen in the left breast. The breast implants were intact.

A few days later, the patient underwent removal of the left implant through the original inframammary incision site. During surgery, approximately 250 ml of serous fluid was drained. A biochemical study showed the following: glucose 1.7 mmol/L, proteins 0.6 g/Dl, and LDH 254 UI/L pH 7.45. The culture was negative. The breast implant was intact with capsule stage Baker I-II.

## Discussion

Silicone breast implants have been used for breast reconstruction and cosmetic enhancement of the breast since the early 1960s and is a frequently performed procedure in the field of plastic surgery. Complications are uncommon but do occur. The implantation of silicone prostheses lead to early and late complications. The most common early complications are infection and hematoma formation. The incidence of periprosthetic hematoma in the immediate postoperative period ranges from 2% to 10.3%, and usually occurs within the first 3 days [3,4]. Among late complications, the most common is capsular contracture. Although late hematoma and seroma are extremely rare complications, reports relating to these breast augmentation complications are increasing. There are no data regarding its incidence in the literature and only sporadic reports are found in the literature. However many incidences are not reported, and the true rate of occurrence of these complications is unknown.

Although there is no clear etiology for seromas, suggested causes include vigorous activity, mechanical trauma including tight squeezing, and chronic inflammatory reaction to the polyurethane-coated implants. Late seroma is thought to be exclusively a complication of textured implants. Irritation of the surrounding tissue by the roughened shell surface, which enhances fluid exudation, is thought to play a role in seroma formation, although there is no experimental evidence to confirmed this [5,6]. Postoperative hematoma and seroma may contribute to infection and/or capsular contracture. No identifiable etiology could be found in our case.

Physical examination is not sensitive enough for the differential diagnosis between rupture of the silicone gel implant or other complications, such as hematoma or seroma. The “gold standard” for confirmation of rupture is explantation and inspection of the implant. However, MRI is a reliable modality for the detection of silicone, and provides an excellent overview of the breast, implant, axilla, and chest wall. MRI has a high capability for differentiating soft tissue masses and allows perfect distinction between simple fluid collections, hematoma, soft tissue masses, fibrosis, and free silicone as a result of prosthesis rupture. In addition, the anatomic mapping is easily communicated to surgeons [4].

However, patients must lie prone for the examination, which can be an uncomfortable position. MRI is also expensive, costing at least three times more than ultrasound.

Many small seromas resolve spontaneously without intervention. Larger seromas that produce problematic asymmetry require open evacuation. Although it is possible to drain seromas percutaneously with ultrasound guidance, there is small risk of damaging the implant. The safest course is to insert a vacuum drain under direct visualization and sterile conditions [7,8].

## Conclusions

Late seroma occurring after aesthetic breast augmentation with textured silicone prosthesis is a very rare complication. Clinicians should include periprosthetic fluid collections in the differential diagnosis of enlarged breast after augmentation. There is no definitive theory about their etiology or any suggestion on how to avoid them.

Imaging examinations, particularly MRI, play an important role in the diagnosis. It is important for radiologists to know the MRI findings of this complication, which suggest the correct diagnosis, to avoid unnecessary additional procedures.
